# Supplementary material for: Identification and Characterization of a Rare Exon 22 Duplication in CFTR in Two Families
Source: Int J Mol Sci. 2025 May 8;26(10):4487. doi: 10.3390/ijms26104487 (PMC12110946; doi:10.3390/ijms26104487)
Supplement: Supplementary file 1 [file ijms-26-04487-s001.zip › Supplementary Material_Revised.pdf]

Supplementary Material

Table S1: Primer-pairs used for bidirectional DNA Sanger-Sequencing of both probands

Primer	Sequence 5'-3'	Genomic position (GRCh38) on Chr7	Exon	Length of PCR product in bp
Ex 11 Forward	GCAGAGTACCTGAAACAGGA	117,559,300 - 117,559,319	11	491
Ex 11 Reverse	CATTCACAGTAGCTTACCCA	117,559,771 – 117,559,790		
Ex 22 Forward	GCCCGACAAATAACCAAGTGA	117,627,394 – 117,627,414	22	454
Ex 22 Reverse	GCTAACACATTGCTTCAGGCT	117,627,827 – 117,627,847		

Table S2: RNA-Primer used for bidirectional Sanger-Sequencing

Primer	Sequence 5'-3'	cDNA position on NM_000492.4	Exon	Length of PCR product in bp
RNA Ex22B Forward	TGCAGTGGGCTGTAAACTCC	3428 - 3447	21-23	321
RNA Ex22B Reverse	TCCCTGATCCAGTTCTTCCCA	3728 - 3748		

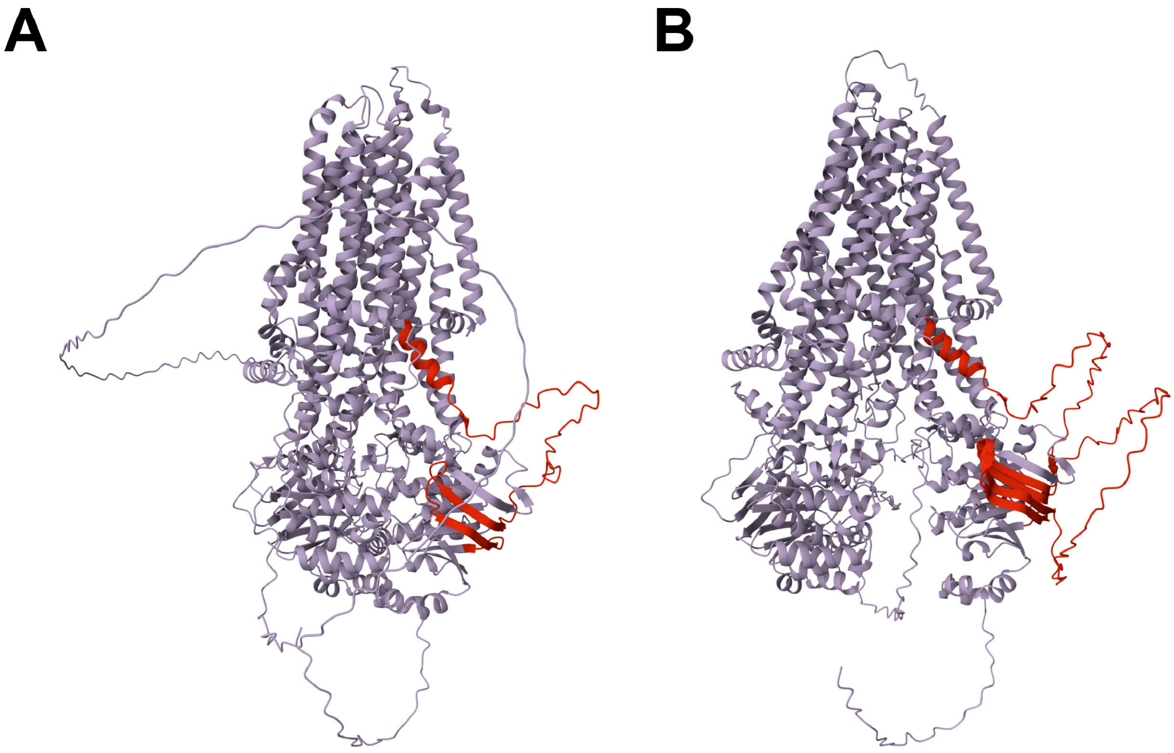

Figure S1: Structure prediction using AlphaFold 3 [1]; **A** predicted structure of the wild type CFTR protein using the canonical *CFTR* sequence (1480 aa), in red exon 22 (aa 1157-1239); predicted template modeling (pTM) = 0.74 **B** predicted structure of the CFTR protein (1563 aa), elongated by an additional exon 22 sequence adjacent to aa 1239, in red exon 22 sequences (aa 1157-1321); pTM = 0.7.

References

1. Abramson, J.; Adler, J.; Dunger, J.; Evans, R.; Green, T.; Pritzel, A.; Ronneberger, O.; Willmore, L.; Ballard, A.J.; Bambrick, J.; et al. Accurate structure prediction of biomolecular interactions with AlphaFold 3. *Nature* **2024**, *630*, 493–500, doi:10.1038/s41586-024-07487-w.
